# Separation of Plasma from Whole Blood by Use of the cobas Plasma Separation Card: a Compelling Alternative to Dried Blood Spots for Quantification of HIV-1 Viral Load

**DOI:** 10.1128/JCM.01336-18

**Published:** 2019-03-28

**Authors:** Sergio Carmona, Britta Seiverth, Dieketseng Magubane, Lucia Hans, Matthias Hoppler

**Affiliations:** a University of the Witwatersrand, Faculty of Health Sciences, School of Pathology, Department of Molecular Medicine and Haematology, Johannesburg, South Africa; b National Health Laboratory Service, Johannesburg, South Africa; c Roche Diagnostics International AG, Rotkreuz, Switzerland; Rhode Island Hospital

**Keywords:** HIV, viral load, cobas, dried blood spots, filter paper cards, HIV patient monitoring, plasma, plasma separation card

## Abstract

Plasma HIV viral load testing is the preferred means of monitoring antiretroviral treatment response. Dried blood spots (DBSs) hold considerable logistical advantages over EDTA samples, but they more frequently misclassify virological failure and have higher limits of detection (LoD). Plasma separation cards (PSCs) may overcome these limitations. Health workers collected EDTA whole blood by venipuncture and 140 μl of finger-prick blood by capillary tube from 53 HIV-infected adults. Capillary blood was immediately transferred to PSCs. Additionally, 432 EDTA samples from HIV-infected adults were spotted onto PSCs and analyzed together with the finger-prick samples. Specificity and sensitivity of PSC with paired EDTA-PSC samples tested on a cobas 6800/8800 system with the cobas HIV-1 test (cobas HIV) was determined. LoD (3rd HIV-1 WHO International Standard) and stability at a range of temperatures and storage durations was determined using cobas HIV and cobas AmpliPrep/cobas TaqMan HIV-1 test v2.0 (CAP/CTM). Of 132 specimens with quantitative values for paired EDTA-PSC samples, the mean log_10_ difference between samples was 0.05 copies/ml (95% confidence interval [CI], −0.01 to 0.11). The LoD for cobas HIV was 790.2 copies/ml and for CAP/CTM was 737.9 copies/ml. At 1,000 copies/ml, PSC sensitivity was 97.0% (128/132) and specificity was 97.2% (343/353). Results correlated well with those from EDTA samples (Deming *R*^2^ = 0.90). PSC results were unaffected by temperature and storage conditions. PSC samples correlate well with plasma viral load and have adequate sensitivity and specificity. The improved performance may be as a result of a reduction in contribution from cell-associated viral nucleic acids. The card provides an alternative sample collection technology to DBSs.

## Abstract (precis)

Plasma HIV viral load testing is the preferred means of monitoring antiretroviral treatment response. Dried blood spots (DBSs) hold considerable logistical advantages over EDTA samples, but they more frequently misclassify virological failure and have higher limits of detection (LoD).

## INTRODUCTION

Presently, of the estimated 36.7 million people infected with HIV worldwide, about 20 million are receiving antiretroviral treatment (ART) (1). Those who are not yet receiving ART are eligible for treatment regardless of their CD4 cell count or clinical status, and concerted efforts are under way to ensure these patients are identified and commence treatment. Once treatment begins, patients require careful monitoring of their responses. Viral load is the most reliable monitoring tool and is preferred over clinical assessment or immunological monitoring through CD4 enumeration (2). The WHO recommends viral load testing biannually in the first year of treatment and annually thereafter (3). Although viral load testing is increasingly becoming available, only about half of patients worldwide who are receiving ART have access to these tests (1).

The timely detection of treatment failure allows health workers to provide additional adherence support for patients or to make changes in treatment regimen (4, 5). In the absence of viral load monitoring, treatment failure is often missed, resulting in HIV disease progression, the accumulation of HIV drug resistant strains, and higher risks of further HIV transmission (6–8). In addition, inadequate access to viral load testing makes it difficult to monitor the overall impact of ART programs, including measurement of progress toward the “third 90”—the goal that 90% of people on ART achieve viral suppression (9).

While plasma is the preferred sample type, viral load measurement from plasma is currently challenging in many settings. Challenges are especially marked in rural areas due to the logistics of transporting plasma samples to centralized testing facilities and the need for sophisticated laboratory infrastructure, qualified personnel, and stringent storage requirements to maintain sample integrity (10). One potential alternative to plasma samples has been the spotting of whole blood from a finger prick onto filter paper that is allowed to dry (11–13). This “dried blood spot” (DBS) facilitates the decentralization of specimen collection while maintaining high throughput at centralized laboratories where blood is eluted off the card for testing (3). The DBS is easy to collect, does not require phlebotomy, minimizes infection risks, and can stabilize biological material at ambient temperature during transport and storage. There are a range of applications of DBSs within the HIV field, including testing for viral resistance and antiretroviral drug levels (14–16), diagnosis of pediatric HIV infection, self-collection of samples for HIV self-testing (17), and population surveillance, including for HIV incidence and for measuring the impact of HIV treatment programs (18–20). Outside of the HIV field, the DBS sample type is well known for enabling the measurement of glucose (21), screening newborns for inherited metabolic disorders, and recent large population-based surveys of infectious diseases, such as hepatitis B and C virus (22).

Although DBSs present many compelling advantages and are included in WHO recommendations for measuring HIV-1 viral loads (3), they have several limitations (3). Most notably, DBS samples can yield higher HIV viral load measurements than plasma-based methods because proviral DNA and intracellular RNA found in whole blood contribute to the HIV molecular signal (23). Viral load testing using DBS may thus misclassify patients as having failed therapy, necessitating additional laboratory testing and even unnecessary switching of treatment regimens. At the same time, there is a concern that DBS samples containing only 50 to 100 μl of whole blood may be suboptimal for adequate representation of the viral population, with inadequate lower limits of viral detection and false-negative results (5, 24).

Innovations in sample collection matrices that overcome the limitations of DBSs could help address the large need for accurate viral load measures (25). This study examined the performance of a novel plasma collection device, the cobas plasma separation card (PSC), which shares many features of the DBS but which collects plasma rather than whole blood. Using paired EDTA plasma and PSC samples, we examined the card’s bias and misclassification at a viral load threshold of 1,000 copies/ml, as well as its usability, limits of detection (LoD), and stability. A successful PSC approach would retain the sample collection and transport advantages of the DBS but have improved sensitivity, specificity, and reliability.

## MATERIALS AND METHODS

The sensitivity component of the study was performed in the Development Laboratory at Roche Molecular Diagnostics International, Rotkreuz, Switzerland, from July to August 2017. All other study components were done in Johannesburg, South Africa, from May to September 2017. The Human Research Ethics Committee (Medical) of the University of the Witwatersrand (no. M160617) and the District Research Committee of Johannesburg approved the study. The health workers and laboratory technicians in the usability assessment gave written informed consent, as did patients who provided blood samples. Test results were not used for patient care. All analyses were done using SAS JMP v12.

### Description of the plasma separation card.

The cobas plasma separation Card is a sampling device that is spotted with whole blood samples obtained from finger pricks or venous blood (140 μl blood per spot). A porous membrane allows only plasma to pass and to be collected on an underlying polyester fleece. The fleece is impregnated with an RNA-stabilizing reagent, which stabilizes the samples over a range of temperatures, humidity levels, and storage conditions. A defined area of the fleece (“the spot”) is then removed and subjected to an elution step before the obtained sample is loaded on either the cobas 6800/8800 system using the cobas HIV-1 test (cobas HIV) or the cobas AmpliPrep/cobas TaqMan HIV-1 test, v2.0 (CAP/CTM).

### Usability assessment.

Participants were recruited from three settings for the usability assessment, conducted per international guidelines (26, 27). First, six phlebotomists collected PSC finger-prick samples at an outpatient clinic of Charlotte Maxeke Johannesburg Academic Hospital (CMJAH), Johannesburg, South Africa. Thereafter, the PSC and its workflow were presented to twelve nurses and one clinic manager drawn from four primary care clinics. Finally, five technologists and medical scientists at the National Health Laboratory Service (NHLS) at CMJAH assessed the preparation and processing of the PSC prior to testing with cobas HIV. In all assessments, participants completed a Likert-type questionnaire covering usability statements for the PSC and its associated workflows.

### PSC performance assessment: sample collection and preparation.

A study nurse collected finger-prick blood samples using a safety lancet (MiniCollect safety lancet, with a penetration depth of 2.0 mm; Greiner Bio-One) from HIV-infected adults attending the HIV clinic at CMJAH (*n* = 53). The nurse collected blood using an EDTA-coated capillary tube positioned directly beneath the puncture site, and 140 μl of blood was transferred onto each of three delineated areas of the card. Venous whole blood samples were then collected by venipuncture using EDTA tubes. We also prepared PSCs using leftover whole blood samples from HIV-infected patients who had recently had a CD4 cell count (*n* = 185) or viral load test (*n* = 241).

**FIG 1:**
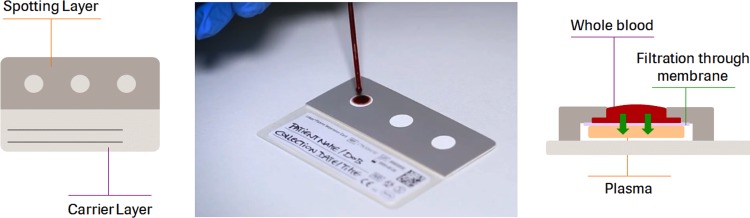
cobas plasma separation card showing the three spots (left) and how plasma is separated from whole blood in the spot (right).

At the NHLS laboratory, the PSCs were placed in a single-use drying rack for a minimum of 4 h at room temperature. PSCs were then packed in separate gas-impermeable zip lock bags with a desiccant and stored at room temperature. In a laminar flow hood, the top layer of the PSC (the “spotting layer”), together with the membrane, was manually removed by pulling on a removable flap on the PSC and then discarded (Fig. 1). A single spot was removed with tweezers and transferred into a cryogenic tube (Cryo.sTM, 5 ml, PP, round bottom; Greiner Bio-One), to which 1,300 μl of cobas specimen preextraction reagent was added. Tubes were placed in a preheated thermomixer (Eppendorf 5355 Thermomixer R with Thermoblock for 24 cryotubes) with 1.5 ml Thermoblock and incubated for 10 min at 56°C and at 1,000 rpm. Thereafter, the tubes were uncapped and loaded into the system for testing. Whole blood in EDTA tubes was centrifuged to generate plasma.

Plasma samples (EDTA-plasma and PSC) were analyzed in single determinations using cobas HIV. Handling of systems, specimens, controls, and reagents was carried out according to procedures described in the cobas 8800 Systems User Guide, Publication version 3.0, software version 1.2. Execution of the study was performed using one cobas 8800 system. A single lot of PSC and a single lot of cobas specimen preextraction reagent were used. Assessments of the limits of detection (LoD) and sample stability were tested with both the cobas HIV and CAP/CTM assays. The cobas HIV LoD is 13.2 copies/ml, and the lower limit of quantification (LLOQ) is 20 copies/ml in plasma, using 0.5 ml of sample input volume. The CAP/CTM has an LoD of 16.5 copies/ml and an LLOQ of 20 copies/ml, using 0.85 ml of sample input volume. PSC results were not adjusted for hematocrit level.

### PSC performance assessment: equivalency, sensitivity and specificity.

To determine matrix (plasma type) equivalency, results from EDTA and PSC samples were compared and tested by cobas HIV. Samples with large differences between the viral load from EDTA and by PSC were also tested with CAP/CTM. We calculated the log_10_ viral load difference and the mean deviation between matched samples. All valid results with a quantitative value of >0 copies/ml in both matrices (EDTA and PSC) were included in the equivalency calculation. A linear regression analysis using Deming regression was also conducted.

Test results generated for the matrix equivalency testing were analyzed to determine the sensitivity and specificity at a viral load threshold of 1,000 copies/ml (WHO recommended threshold for treatment failure) (3).

### PSC performance assessment: limits of detection and stability assessments.

Three independent dilution series of five concentration levels for HIV-1 (plus a blank control) were prepared by diluting positive spike-in panel members (HIV-1 group M subtype B from MVP899 cell culture supernatant) 1:100 in HIV-negative EDTA whole blood. The study panels were pipetted onto three different PSCs and multiple replicates tested using cobas HIV and CAP/CTM. A part of the first panel member was centrifuged and titer assigned by the calibrator bracketing method using cobas HIV with the 3rd HIV-1 International Standard (WHO). Probit values were determined at the 95% hit rate, together with the lowest panel member with at least a 95% reactive rate.

To assess stability, PSCs were prepared using HIV-negative whole blood that had been spiked with HIV-1 at approximately five times the LoD. Viral load was measured in control PSC, with the remainder evaluated after being stored for various time periods at temperatures ranging from −10°C or lower to 45°C and at a humidity of 85%.

## RESULTS

### Usability.

**FIG 2:**
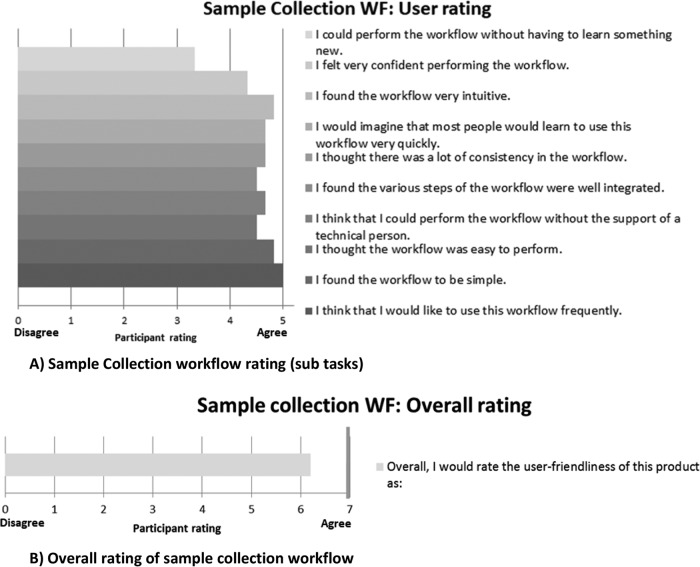
Usability assessment of sample collection workflow.

The phlebotomists rated the sample collection positively, scoring most subtasks above 4 out of 5 (“agree”) (Figs 2A and B). The lancet, capillary tube, and workflow were considered user friendly. However, the phlebotomists—who had not been trained—had some difficulties obtaining sufficient samples, determining when the capillary was adequately filled with blood, obtaining samples without air bubbles, and applying the correct amount of blood to the PSC. Of the 51 capillaries collected, 5 clotting events occurred, due to air bubbles in the tube or delays in spotting the blood on the PSC. Once these phlebotomists became familiar with the use of capillary tubes, their performance improved markedly. Health care workers participating in the study believed that the PSC would be very useful in their setting. A few health workers believed that the sample collection workflow did not appear user friendly, mainly due to the use of a capillary and the volume of blood to be collected.

Laboratory staff rated all the subtasks in the preanalytical workflow assessment as above 4 (“agree”). A few errors occurred on isolation of the spot and application, in which the filtration membrane was transferred rather than the spot. Overall, however, across all components of the usability assessment, no critical product risks were identified.

### Equivalency, sensitivity, and specificity.

A total of 535 PSCs were processed, of which 23 yielded invalid results (4.3%), 9 were duplicates (1.7%), 6 had no paired plasma viral load results (1.1%) and 8 had no HIV-positive status confirmed (1.5%). Of the 521 EDTA-plasma samples processed, 19 were invalid (3.6%). A further 13 samples were excluded; 1 was a duplicate, 4 had no paired PSC result, and 8 had no HIV-positive status confirmed. Additionally, 4 tests on both PSC and EDTA-plasma were excluded due to operator errors.

**FIG 3:**
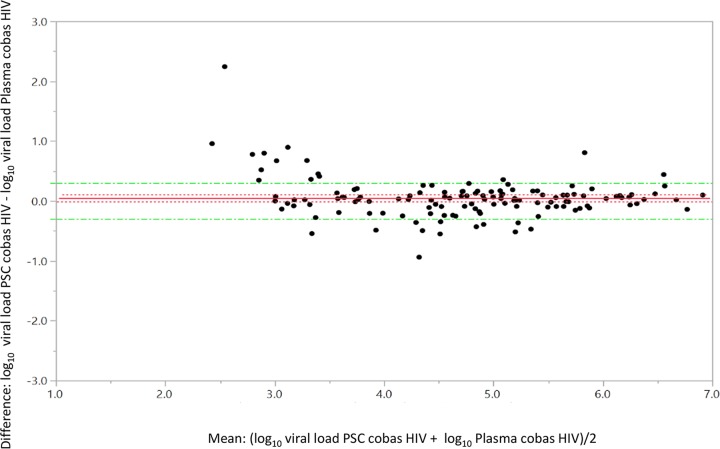
Bland-Altman graph for matrix (plasma type) equivalency with the cobas HIV-1 test, showing the difference in matched pairs in log_10_ titer PSC-log_10_ titer plasma (mean difference, 0.05; standard error, 0.03; upper 95%, 0.11; lower 95%, −0.01; *n* = 132. A total of 353 samples did not have a quantifiable result on both matrices; 333 were target not detected, 13 were below the limit of detection, and 7 were above the limit of detection).

**FIG 4:**
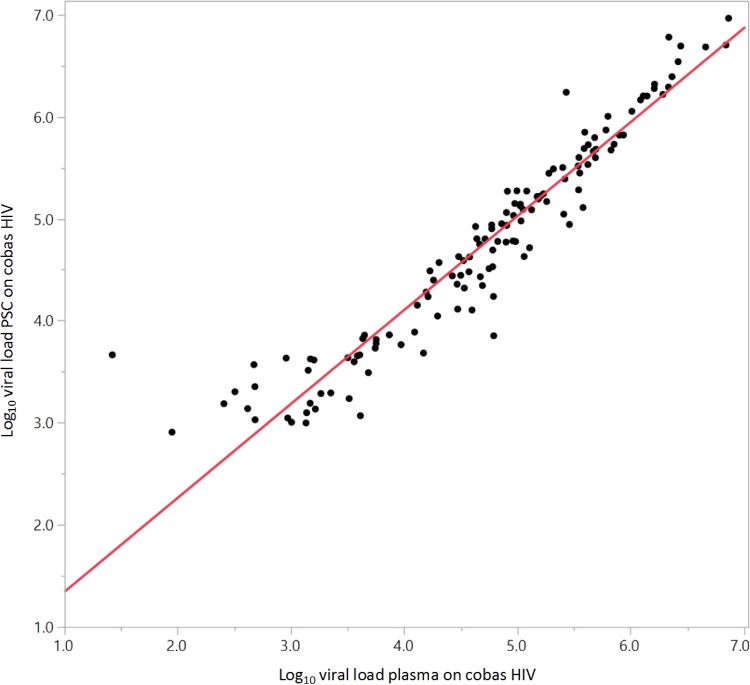
Deming regression for matrix (plasma type) equivalency with the cobas HIV-1 test.

A total of 485 samples had valid paired EDTA and PSC results; of these, 132 samples had quantifiable results for both matrices. The overall mean log_10_ difference in titer between EDTA-plasma and PSC-plasma was 0.05 (95% confidence interval [CI], −0.01 to 0.11; Fig. 3). The Deming regression analysis line slope was 0.92, the *y* intercept was 0.42, and *R*^2^ was 0.90 (Fig. 4).

Analysis of all 485 paired EDTA and PSC samples at a 1,000 copies/ml threshold using the cobas HIV yielded a sensitivity of 97.0% (128/132; 95% CI, 92.4 to 99.2%) and specificity of 97.2% (343/353; 95% CI, 94.9 to 98.6%). In the 14 specimens with discordant EDTA and PSC results, almost all had a viral load of around 1,000 copies/ml, with a median log_10_ titer of 3.13 (interquartile range [IQR] = 3.00 to 3.35). The median log_10_ difference between the PSC and plasma viral load for the misclassified specimens was 0.60 (IQR = −0.14 to 0.80). No differences in findings were noted by sample type.

### Limits of detection and stability.

The viral load of 63 replicates per concentration level were measured in the LoD assessment (Table 1). Based on probit analysis for all PSC lots combined, the LoD was 790.2 copies/ml with cobas HIV (95% CI, 658.9 to 1,003.6 copies/ml) and 737.9 copies/ml with CAP/CTM (95% CI, 614.3 to 938.5 copies/ml). For each of the storage conditions listed in Table 2, there was a 100% detection rate for HIV. The mean difference between the stored specimens and the controls was <0.2 log_10_ copies/ml for all the stability conditions assessed.

**TABLE 1:** Limits of detection of the plasma separation card for cobas HIV^*b*^

Concn (copies/ml)	No. of valid samples^*a*^	No. of reactive samples	Detection rate (%)
1,971.1	62	62	100.0
1,925.3	63	63	100.0
1,358.1	62	62	100.0
985.5	63	62	98.4
962.6	63	62	98.4
679	63	59	93.7
657	63	59	93.7
641.8	63	57	90.5
452.7	63	52	82.5
328.5	63	47	74.6
320.9	63	46	73.0
226.3	62	49	79.0
164.3	63	36	57.1
160.4	63	35	55.6
113.2	63	38	60.3
0	63	0	0.0
0	63	0	0.0
0	63	0	0.0

a Some sample sizes were <63 due to an insufficient number of spots. Data for limit of detection analysis for CAP/CTM are not shown.

b Limit of detection by probit analysis (95% hit rate), 790.2 copies/ml. 95% confidence interval (CI), 658.9 to 1,003.6 copies/ml. Lowest panel member with ≥95% reactive rate, 962.6 copies/ml 98.4% (62/63); 95% CI = 91.5 to 100.0%; lower one-sided 95% CI = 92.7%.

**TABLE 2:** Clinical specimen stability results

Time point	Storage conditions (no. of days, temperature [°C], relative humidity [%])^*a*^	Layer separation after 21 days (transport stability)^*b*^	Valid replicates	HIV-1 reactive rate (%)	Mean log_10_ titer	Mean log_10_ titer difference from that at T0
1	T0	NA	12/12^*c*^	100	3.45	0.00
2	21, 45, 85	NA	15/15	100	3.42	−0.03
3	21, 45, 85 + 28, 18–30	No	15/15	100	3.48	0.03
	21, 45, 85 + 28, 2–8	No	15/15	100	3.38	−0.07
	21,45, 85 + 28, ≤10	No	14/14	100	3.46	0.01
4	21, 45, 85 + 35, 18–30	Yes	14/14	100	3.35	−0.10
	21, 45, 85 + 35, 2-8	Yes	15/15	100	3.43	−0.02
	21, 45, 85 + 35, ≤10	Yes	15/15	100	3.46	0.01
5	21, 45, 85 + 56, 18–30	No	15/15	100	3.35	−0.09
	21, 45, 85 + 56, 2–8	No	15/15	100	3.42	−0.02
	21, 45, 85 + 56, ≤10	No	15/15	100	3.37	−0.08
	21, 45, 85 + 56, 18–30	Yes	14/14	100	3.48	0.03
	21, 45, 85 + 56, 2–8	Yes	15/15	100	3.45	0.00
	21, 45, 85 + 56, ≤10	Yes	15/15	100	3.43	−0.02

a T0, time of initial measurement.

b n/a, not applicable.

c Could not repeat as no additional T0 samples were available.

## DISCUSSION

The PSC performed well in measures of equivalency, LoD, and sample stability. Most importantly, the mean deviation of the log_10_ viral load between PSC and matched EDTA-plasma specimens was 0.05 log10, suggesting that the card is adequate for use in the monitoring of HIV viral load. The rate of upward misclassification of results, where specimens are incorrectly categorized as being above 1,000 copies/ml or virological failure, was 3%. This is considerably lower than that in many reports of DBS analyses, where upward misclassification rates can reach 10 to 15% or higher (25, 28). The LoD of around 750 copies/ml with cobas HIV and with CAP/CTM is considerably higher than that with EDTA specimens on those systems (around 20 copies/ml).

Even with the introduction of point-of-care viral load devices, it is expected that DBS and related technologies, such as the PSC, will remain relevant tools for expanding access to viral load monitoring. This is especially true in rural settings, but also even in some of the urban clinics visited in this study. There is clearly a need, however, for optimizing the performance of DBS technologies. The impact of interference of cell-associated viral nucleic acids in viral load measures is highest when plasma viral load is low, as in patients successfully treated with ART. Indeed, most DBSs perform poorly at thresholds of around 1,000 copies/ml (25, 29–31). Several DBS protocols have been designed to minimize the contribution of cell-associated viral nucleic acid by selectively purifying RNA during sample preparation or by preferentially eluting plasma-associated virus from a DBS, with some success (32, 33). The PSC described in this paper presents a novel, alternative strategy for overcoming this vexing problem.

The stability of the PSC is reassuring, indicating that a specimen collected with the PSC can be transported for at least 28 days and then stored for 56 days at a wide range of temperatures without compromising the HIV viral load result. Although DBS specimens generally have high levels of stability (34–36), some evidence suggests that caution is still required in this regard (37). Overall, the sample collection and the preanalytical workflow of the PSC appear to be of low complexity and user friendly, especially once staff become familiar with the procedures. The usability findings, however, highlighted areas that need to be foregrounded in the training and documentation that accompany the card. These mostly concern the correct use of capillary tubes to avoid blood clots and to ensure correct spotting of the PSC. Rates of invalid samples linked to the PSC specimens were around 4% and would likely reduce as health workers become more proficient in sample collection. Health workers are increasingly familiar with collecting DBS samples, and this expertise would likely be transferable to PSC use. Having multiple spots on the PSC is a strength of the card, allowing for repeat testing or for additional tests to be done. Collection of three samples of relatively high volume, however, may raise the likelihood of blood clots and pose challenges for health workers, especially with pediatric patients.

The study had several limitations. Most notably, it was conducted in a single urban center, distinct from many of the settings where the card would be used. Direct comparison between the PSC and DBS cards under field conditions would assist countries in selecting the optimum approach for their circumstances. To date, many of the studies that measure the performance of DBS and related technologies in field conditions are of poor quality (38). Also, it is necessary to confirm the performance of PSCs in other assays, such as those for HIV viral resistance, drug level monitoring (e.g., ART), and for diagnosing hepatitis B and C viral infections.

In conclusion, a technology that allows for the collection of plasma using finger-prick specimens would retain the sample collection and transport advantages of a DBS but produce plasma as the testing matrix; this is a major development for the field. The findings presented here on the performance and usability of the PSC are promising. This technology supports the efforts to scale up viral load testing, which is essential for ART monitoring, and indeed, most importantly, for the achieving of the “third 90.” Success of the ongoing global rollout of antiretroviral treatment depends in no small measure on the levels of access to and quality of tests for monitoring of viral loads.
